# Assembly and comparative analysis of the complete multichromosomal mitochondrial genome of *Cymbidium ensifolium*, an orchid of high economic and ornamental value

**DOI:** 10.1186/s12870-024-04962-4

**Published:** 2024-04-09

**Authors:** Baoming Shen, Airong Shen, Lina Liu, Yun Tan, Sainan Li, Zhuming Tan

**Affiliations:** Institute of Forest and Grass Cultivation, Hunan Academy of Forestry, 658 Shaoshan South Road, Tianxin District, Changsha City, 410004 China

**Keywords:** *Cymbidium ensifolium*, Multichromosomal mitochondrial genome, Comparative analysis, Repeat sequence

## Abstract

**Background:**

Orchidaceae is one of the largest groups of angiosperms, and most species have high economic value and scientific research value due to their ornamental and medicinal properties. In China, Chinese *Cymbidium* is a popular ornamental orchid with high economic value and a long history. However, to date, no detailed information on the mitochondrial genome of any species of Chinese *Cymbidium* has been published.

**Results:**

Here, we present the complete assembly and annotation of the mitochondrial genome of *Cymbidium ensifolium* (L.) Sw. The mitogenome of *C. ensifolium* was 560,647 bp in length and consisted of 19 circular subgenomes ranging in size from 21,995 bp to 48,212 bp. The genome encoded 35 protein-coding genes, 36 tRNAs, 3 rRNAs, and 3405 ORFs. Repeat sequence analysis and prediction of RNA editing sites revealed a total of 915 dispersed repeats, 162 simple repeats, 45 tandem repeats, and 530 RNA editing sites. Analysis of codon usage showed a preference for codons ending in A/T. Interorganellar DNA transfer was identified in 13 of the 19 chromosomes, with plastid-derived DNA fragments representing 6.81% of the *C. ensifolium* mitochondrial genome. The homologous fragments of the mitochondrial genome and nuclear genome were also analysed. Comparative analysis showed that the GC content was conserved, but the size, structure, and gene content of the mitogenomes varied greatly among plants with multichromosomal mitogenome structure. Phylogenetic analysis based on the mitogenomes reflected the evolutionary and taxonomic statuses of *C. ensifolium*. Interestingly, compared with the mitogenomes of *Cymbidium lancifolium* Hook. and *Cymbidium macrorhizon* Lindl., the mitogenome of *C. ensifolium* lost 8 ribosomal protein-coding genes.

**Conclusion:**

In this study, we assembled and annotated the mitogenome of *C. ensifolium* and compared it with the mitogenomes of other Liliidae and plants with multichromosomal mitogenome structures. Our findings enrich the mitochondrial genome database of orchid plants and reveal the rapid structural evolution of *Cymbidium* mitochondrial genomes, highlighting the potential for mitochondrial genes to help decipher plant evolutionary history.

**Supplementary Information:**

The online version contains supplementary material available at 10.1186/s12870-024-04962-4.

## Background

Orchidaceae is a family of perennial flowering plants and one of the world’s largest families of angiosperms, with 27,801 species in 899 genera recorded worldwide (http://www.theplantlist.org/1.1/browse/A/Orchidaceae/) (accessed on 16 October 2023). According to their habitats, orchids can be classified into terrestrial, epiphytic, and saprophytic types, most of which have high ornamental and medicinal value [1, 2]. Orchids have diverse distributions and morphologies and complex genomes [3]. They are also typical mycorrhizal plants, as almost all orchids have symbiotic relationships with fungi. Mycorrhizal symbiosis occurs throughout the entire life cycle of orchids, from seed germination to flowering and fruiting [4]. These characteristics make orchids one of the most evolutionarily derived groups of plants [5]. Current research on orchids primarily focuses on discovering new species, breeding techniques, symbiotic relationships with fungi and other microorganisms, species identification techniques, gene research, chloroplast genomes, and a limited number of genome studies [6–13]. However, research on the mitochondrial genomes of orchids is relatively scarce [14–16]. In particular, no mitochondrial genome data for the Chinese *Cymbidium* species in the *Subgen. Jensoa* (Rafin.) Seth et Cribb, including *Cymbidium sinense* (Jack. ex Andr.) Willd., *Cymbidium faberi* Rolfe, *Cymbidium goeringii* (Rchb. f.) Rchb. F., *Cymbidium kanran* Makino, and *C. ensifolium*, have been published online.

Mitochondria are important organelles in plant cells that participate in many metabolic processes related to the production of ATP energy storage molecules and cytoplasmic male sterility (CMS) [17, 18]. Regarding their origins, the currently recognized hypothesis is the serial endosymbiosis theory, which posits that mitochondria are the direct descendants of a bacterial endosymbiont that became established at an early stage in a nucleus-containing (but amitochondriate) host cell [19]. The mitochondrial genomes of plants have some unique characteristics compared to chloroplast genomes and animal mitochondrial genomes, including a broad distribution in genome size, extensive repeat-mediated homologous recombination, multiple horizontal and intracellular gene transfers, gain or loss of genes or entire chromosomes, a high density of introns within genes, specific trans-splicing associated with different intron groups, RNA editing at the RNA level, and foreign sequence capture [16, 20–25]. However, the sequencing of plant mitochondrial genomes is limited by the easy contamination of chloroplast DNA during mitochondrial DNA extraction [26], as well as the complex structure of the mitochondrial genome. In addition to the single circular structure, mitochondrial genomes can also exist in linear, multiple circular, branched, and complex forms. For example, the mitochondrial genome of *Gastrodia elata* Bl. consists of 19 subgenomes, including 12 circular subgenomes and 7 linear subgenomes [14]. The complete mitochondrial genome of *Picea sitchensis* (Bong.) Carrière is composed of two components: a 168-kb circular segment and a larger 5.36-Mb component composed of 12 segments [27]. All of these characteristics have resulted in only a few plant mitochondrial genomes being properly characterized, fully sequenced, and properly assembled (NCBI database, 351 land plant mitogenomes, 13 July 2022).

The symbiotic relationship between orchids and fungi makes orchids an outstanding candidate for investigating the evolution of mitogenomes. Sinn and Barrett confirmed that the ancestor of orchids acquired an approximately 270-bp fungal mitogenomic region containing three transfer RNA genes through horizontal gene transfer (HGT) [25]. Although the Orchidaceae family contains nearly 28,000 species [2], to date, only a few draft-assembled mitogenomes of Orchidaceae have been reported, including those of *G. elata*, *Gastrodia angusta* S. Chow & S. C. Chen, *Paphiopedilum micranthum* Tang & F. T. Wang, *C. lancifolium*, *C. macrorhizon*, *Apostasia shenzhenica* Z.J.Liu & L.J.Chen, *Epigeneium amplum* (Lindl.) Summerh., and *Phalaenopsis aphrodite* Rchb. f. (NCBI database, [14–16, 24]), which is far from meeting the needs of big data analysis. Therefore, the mitochondrial genome database of orchids urgently needs to be supplemented with new data.

The Chinese *Cymbidium* species have significant economic and ornamental value, with some variants being sold for millions of dollars. The genetic diversity within these species makes them valuable for experimentation. The Chinese *Cymbidium* species belong to the *Cymbidium* genus, specifically *Subgen. Jensoa*, which contains three sections: Sect. Geocymbidium, Sect. Pachyrhizanthe, and Sect. Jensoa. While mitochondrial genomes have been reported for *C. lancifolium* and *C. macrorhizon* [15], there are no such published data available for the economically valuable Chinese *Cymbidium* species in Sect. Jensoa.

In this study, we assembled and annotated the *C. ensifolium* mitogenome for the first time. We also conducted a comprehensive analysis of its characteristics, repetitive sequences, RNA editing, codon preferences, and migration sequences and performed comparative genomics with other Liliidae plants and plants with multichromosomal mitogenome structures. Additionally, we performed a phylogenetic analysis. These results will contribute to a better understanding of the structure and function of the *C. ensifolium* mitogenome and provide useful molecular markers for conservation biology, population genetics, and evolutionary studies of this species.

## Results

### Multichromosomal structure of the *C. ensifolium* mitogenome

**Fig. 1:**
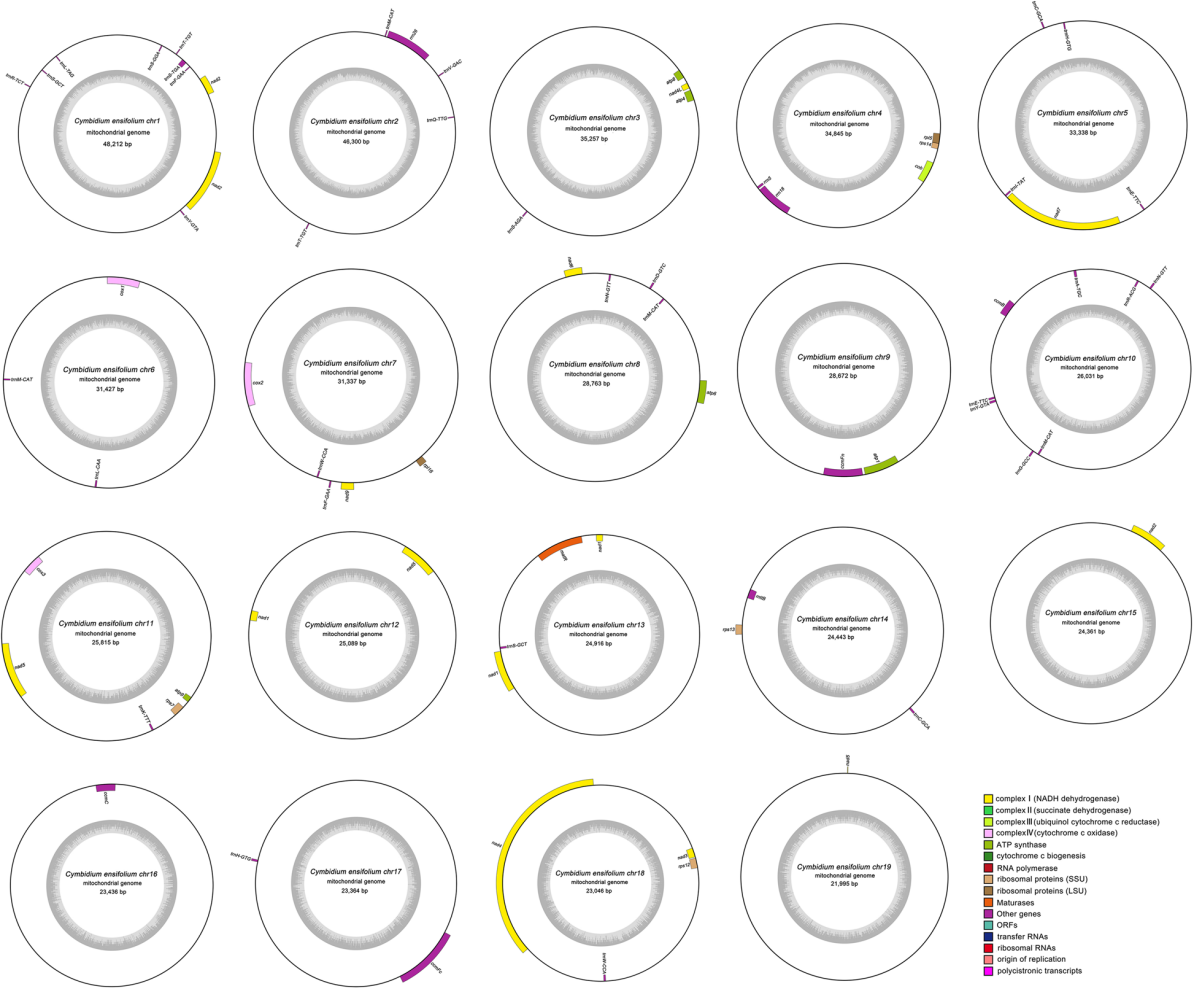
Map of the *C. ensifolium* mitogenome. The mitogenome consists of 19 circular chromosomes. Genes located inside and outside each circle are transcribed clockwise and counterclockwise, respectively. The dark gray region in the inner circle represents the GC content

In our study, the raw data yielded 92.6 G of Illumina sequencing data and 12.5 G of PacBio RSII sequencing data. The average read length was 10,525 bp (Tables S1 and S2). The mitogenome of *C. ensifolium* was assembled into 19 circular chromosomes with lengths ranging from 21,995 bp to 48,212 bp, resulting in a total length of 560,647 bp. The size of the 19 mitochondrial subgenomes was generally average, without a large main circle (Fig. 1). The average guanine-cytosine (GC) content of the *C. ensifolium* mitogenome was 43.89%, ranging between 43.26% and 45.80% among chromosomes (Table S3). The sequencing depth for the majority of the chromosomes was above 300 × for long reads and 1200 × for short reads (Table S4). We annotated a total of 74 genes in the *C. ensifolium* mitogenome, including 35 protein-coding genes (PCGs), 36 tRNA genes, and 3 rRNA genes. A total of 3,405 open reading frames (ORFs) were also identified.

The PCGs could be categorized into 11 groups (Table 1), namely, ATP synthases (5 genes), cytochrome C biogenesis genes (4 genes), ubiquinol cytochrome c reductases (1 gene), cytochrome C oxidases (3 genes), maturases (1 gene), transport membrane proteins, NADH dehydrogenases (9 (14) genes, where nad1 and nad 5 had three copies each and nad2 had two copies), ribosomal proteins (LSU, 2 genes), and ribosomal proteins (SSU, 4 genes). Each submitochondrial genome contained one to four PCGs, except for *C. ensifolium chr2*, which had zero PCGs (Table S3).

**Table 1:** Functional classification of genes and physical location of the *C. ensifolium* mitogenome

Group of genes	Gene name	Length (bp)	Start codon	Stop codon	Amino acid
ATP synthase	*atp1*	1530	ATG	TGA	510
	*atp4*	579	ATG	TGA	193
	*atp6*	969	ATG	TAG	323
	*atp8*	480	ATG	TAA	160
	*atp9*	225	ATG	CGA(TGA)	75
Cytochrome c biogenesis	*ccmB*	621	ATG	TGA	207
	*ccmC*	723	ATG	TAG	241
	*ccmFc*	1317	ATG	CGA(TGA)	439
	*ccmFn*	1707	ATG	TGA	569
Ubiquinol cytochrome c reductase	*cob*	1182	ATG	TGA	394
Cytochrome c oxidase	*cox1*	1584	ATG	TAA	528
	*cox2*	801	ATG	TAG	267
	*cox3*	798	ATG	TGA	266
Maturases	*matR*	1899	ATG	TAG	633
Transport membrane protein	*mttB*	351	ATG	TAG	117
NADH dehydrogenase	*nad1(3)*	(978,978,978)	ACG(ATG)	TAA	326
	*nad2 (2)*	(1509,1509)	ATG	TGA	503
	*nad3*	357	ATG	TAG	119
	*nad4*	1488	ATG	TGA	496
	*nad4L*	303	ATG	TAA	101
	*nad5 (3)*	(2016,2106,2106)	ATG	TAA	672
	*nad6*	747	ATG	TAG	249
	*nad7*	1185	ATG	TAG	395
	*nad9*	573	ATG	TAA	191
Ribosomal proteins (LSU)	*rpl16*	348	ATG	TAA	116
	*rpl5*	546	ATG	TAA	182
Ribosomal proteins (SSU)	*rps12*	378	ATG	TGA	126
	*rps13*	351	ATG	TGA	117
	*rps14*	303	ATG	TAG	101
	*rps7*	447	ATG	TAA	149
Ribosomal RNAs	*rrn18*	1976			
	*rrn26*	3225			
	*rrn5*	119			
Transfer RNAs	*trnA-TGC*	65			
	*trnC-GCA*	(73,71)			
	*trnD-GTC*	74			
	*trnE-TTC*	(73,72)			
	*trnF-GAA*	(73,73)			
	*trnG-GCC*	71			
	*trnH-GTG*	(74,74)			
	*trnI-TAT*	71			
	*trnK-TTT*	73			
	*trnL-CAA*	81			
	*trnL-TAG*	80			
	*trnM-CAT*	(74,74,74,74)			
	*trnN-GTT*	(72,72)			
	*trnQ-TTG*	72			
	*trnR-ACG*	74			
	*trnR-TCT*	72			
	*trnS-AGA*	67			
	*trnS-GCT*	(77,77)			
	*trnS-GGA*	87			
	*trnS-TGA*	85			
	*trnT-TGT*	(73,73)			
	*trnV-GAC*	72			
	*trnW-CCA*	(74,74)			
	*trnY-GTA*	(83,84)			

The numbers after gene names indicate the number of copies

The *C. ensifolium* mitogenome had 3 rRNA genes and 36 tRNA genes. The rRNA genes included *rrn18*, *rrn26*, and *rrn5*, each with one copy. Among the tRNA genes, there were 24 different types, with *trnC-GCA*, *trnE-TTC*, *trnF-GAA*, *trnH-GTG*, *trnN-GTT*, *trnS-GCT*, *trnT-TGT*, *trnW-CCA,* and *trnY-GTA* having two copies and *trnM-CAT* having four copies. Minor sequence differences were observed in the copies between *trnC-GCA*, *trnE-TTC*, and *trnY-GTA* (Table 1).

The lengths of all PCGs, tRNAs, and rRNAs were 33,792 bp, 2,682 bp, and 5,320 bp, respectively, accounting for 6.03%, 0.48%, and 0.95% of the total mitogenome length (Table S3). Among the 11 different genes containing introns, some had one intron (*ccmFc*, *cox2*, *trnA-TGC*, *trnI-TAT*, *trnS-TGA*, and *trnT-TGT*), while others had multiple introns (*nad1*, *nad2*, *nad4*, *nad5*, and *nad7* had 4 introns, and *nad4* had 3 introns*)*.

### Prediction of RNA editing sites

**Fig. 2:**
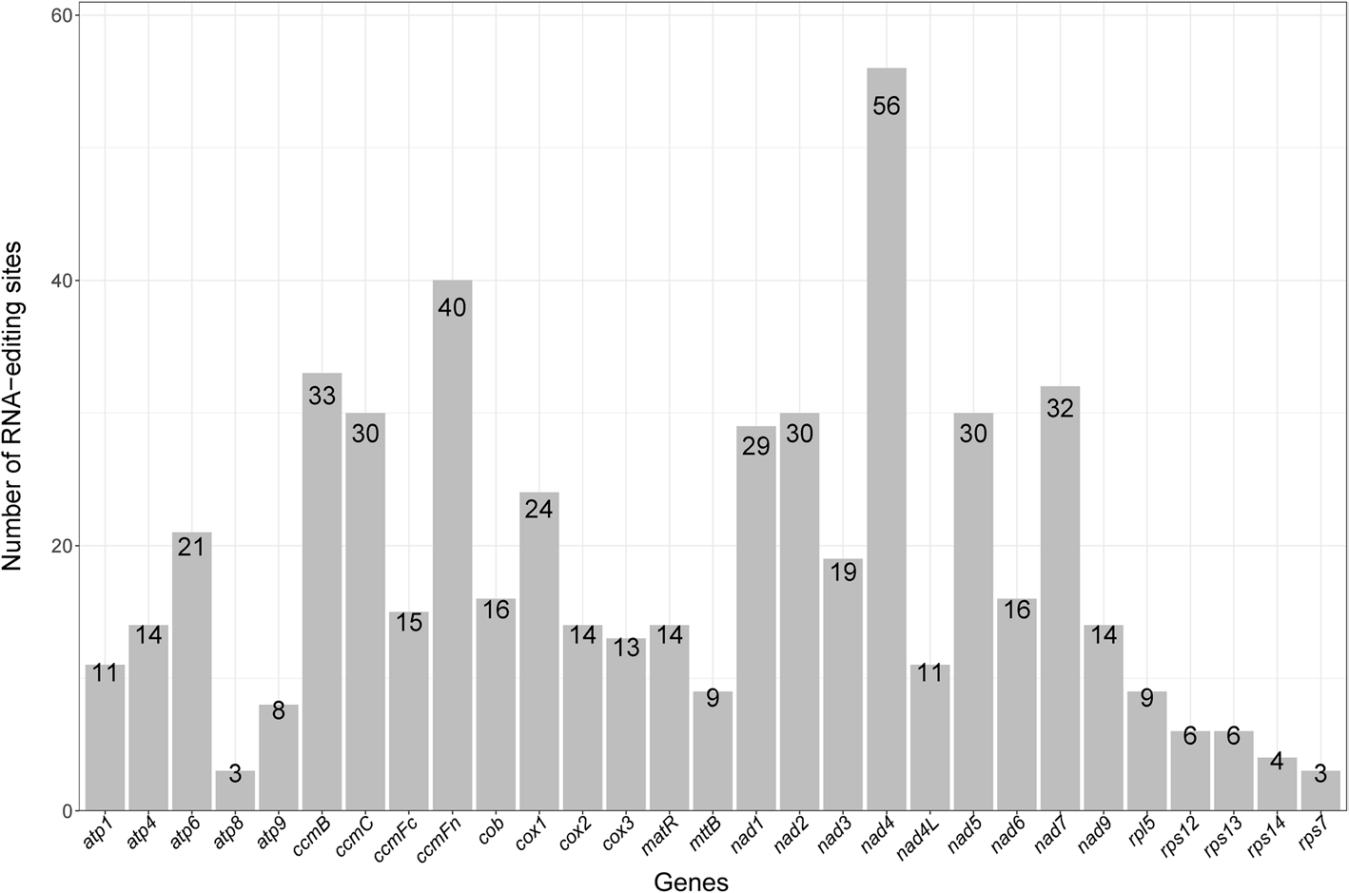
Distribution of RNA editing sites in protein-coding genes of the *C. ensifolium* mitogenome

In the mitochondria of most flowering plants (angiosperms), RNA editing events are common in exon sequences and some noncoding regions and can create more genes than in the corresponding DNA coding sequence [28, 29]. RNA editing sites were predicted in the *C. ensifolium* mitogenome, with a total of 530 sites identified within 29 PCGs (Fig. 2, Table 2). The editing sites were unevenly distributed among the different genes, ranging from 3 (*atp8* and *rps7*) to 56 (*nad4*) sites, except *rpl116*, which had zero RNA editing sites (Fig. 2). After RNA editing, amino acid changes occurred, with 41.70% remaining unchanged in hydrophobicity, 48.68% changing from hydrophilic to hydrophobic, and 9.25% changing from hydrophobic to hydrophilic (Table 2).

**Table 2:** Prediction of RNA editing sites

Type	RNA editing	Number	Percentage
hydrophilic-hydrophilic	CAC (H) = > TAC (Y)	10	
	CAT (H) = > TAT (Y)	18	
	CGC (R) = > TGC (C)	11	
	CGT (R) = > TGT (C)	31	
	total	70	13.21%
hydrophilic-hydrophobic	ACA (T) = > ATA (I)	5	
	ACG (T) = > ATG (M)	7	
	ACT (T) = > ATT (I)	5	
	CGG (R) = > TGG (W)	32	
	TCA (S) = > TTA (L)	76	
	TCC (S) = > TTC (F)	37	
	TCG (S) = > TTG (L)	41	
	TCT (S) = > TTT (F)	55	
	total	258	48.68%
hydrophilic-stop	CGA (R) = > TGA (X)	2	
	total	2	0.38%
hydrophobic-hydrophilic	CCA (P) = > TCA (S)	8	
	CCC (P) = > TCC (S)	14	
	CCG (P) = > TCG (S)	7	
	CCT (P) = > TCT (S)	20	
	total	49	9.25%
hydrophobic-hydrophobic	CCA (P) = > CTA (L)	48	
	CCC (P) = > CTC (L)	10	
	CCC (P) = > TTC (F)	6	
	CCG (P) = > CTG (L)	26	
	CCT (P) = > CTT (L)	26	
	CCT (P) = > TTT (F)	12	
	CTC (L) = > TTC (F)	5	
	CTT (L) = > TTT (F)	9	
	GCA (A) = > GTA (V)	1	
	GCC (A) = > GTC (V)	1	
	GCG (A) = > GTG (V)	4	
	GCT (A) = > GTT (V)	3	
	total	151	28.49%
	All	530	100%

There were only 29 codon transfer types, corresponding to 12 amino acid transfer types. Among all the codon transfer types, the number of types ranged from 1 to 76. GCA and GCC codons had only one site each transferred to GTA and GTC codons, while the TCA codon was the most common, with 76 sites transferred to the TTA codon. The predicted results also indicated a change in the types of amino acids, increasing from seven to ten. Most of the amino acids after the transfer were different from those before. The highest tendency after codon editing was the conversion to leucine, with 42.83% (227 sites) of the amino acids being converted. All RNA editing sites in the *C. ensifolium* mitogenome were of the C-T editing type. Among these sites, all were located at the first two bases of the triplet codon, primarily at the second base with 69.49% (363 sites), while the remaining 31.51% (167) were located at the first base. Additionally, the amino acid CGA (R) was edited to the stop codon TGA (X).

### Analysis of codon usage bias in the *C. ensifolium* mitogenome

The codon composition of the *C. ensifolium* mitogenome was analyzed (Table S5). The total number of codons in all coding genes was 8765, and the GC1, GC2, and GC3 contents and the average GC content of all three bases (all GC) were 49.4%, 43.98%, 38.73%, and 44.04%, respectively. All of these values were less than 50%, indicating a bias in the codons of the *C. ensifolium* mitogenome due to the use of both A and T bases. The effective number of codons (Nc) was 53.48, suggesting a weak codon preference in the *C. ensifolium* mitogenome [30, 31]. As presented in Table 1, all of the PCGs used ATG as the start codon. The utilization rates of the TAA, TGA, and TAG stop codons were 37.14%, 37.14% and 25.71%, respectively, with the TAG stop codon having the lowest usage rate.

**Fig. 3:**
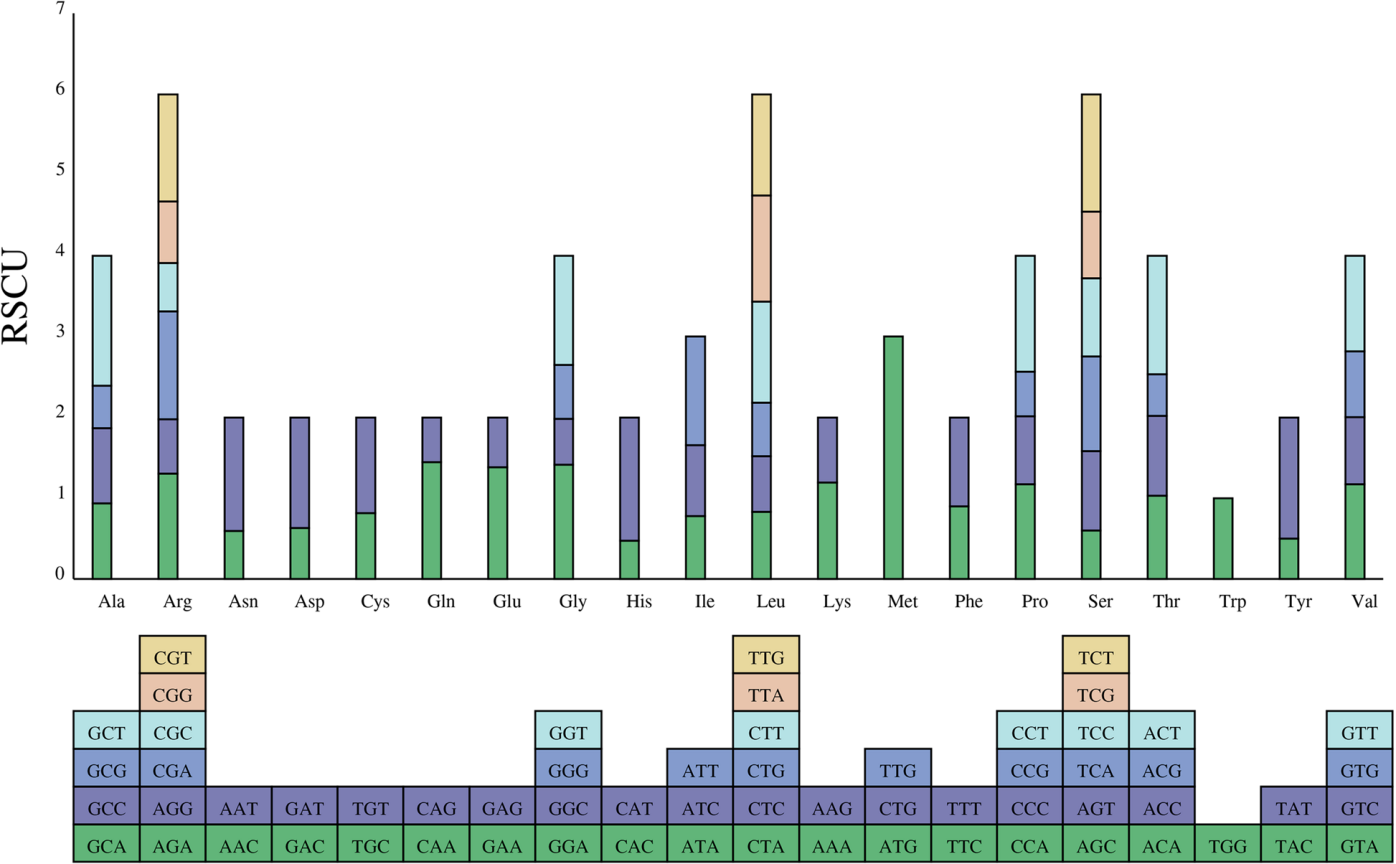
Relative synonymous codon usage (RSCU) in the *C. ensifolium* mitogenome. The x-axis represents different amino acids. RSCU values indicate the observed number of times a particular codon is used relative to the expected number of times that codon would be used based on uniform synonymous codon usage

The relative synonymous codon usage (RSCU) method was used to measure codon usage bias in the *C. ensifolium* mitogenome (Table S5). RSCU can eliminate the influence of amino acid composition on codon usage and directly reflect the differences in codon usage patterns. An RSCU value of 1 indicates unbiased codon usage, while an RSCU value greater than 1 indicates a higher relative frequency of usage and an RSCU value less than 1 indicates a lower usage frequency [32]. The results showed that there were 29 codons with RSCU > 1, indicating that the usage frequency of these codons was higher than that of other synonymous codons. Among these, all the codons ended with the A/T base, except for UUG (1.2546) and AUG (3) (Fig. 3, Table S5).

### Repeat sequences in the *C. ensifolium* mitogenome

**Fig. 4:**
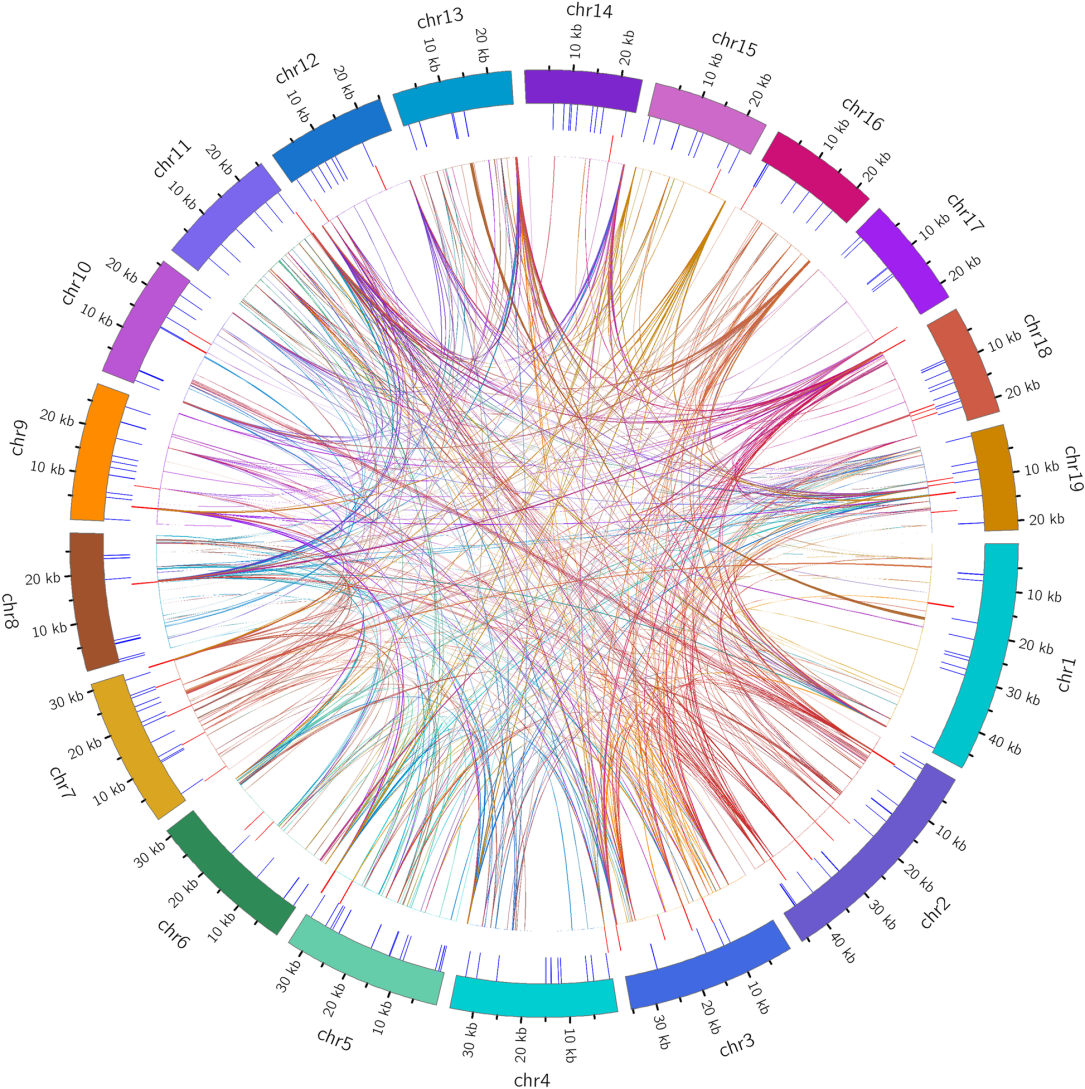
Distribution of repetitive sequences in the *C. ensifolium* mitogenome. The outermost circle represents SSRs, followed by tandem repeat sequences, and the innermost concatenation represents dispersed repeat sequences

**Fig. 5:**
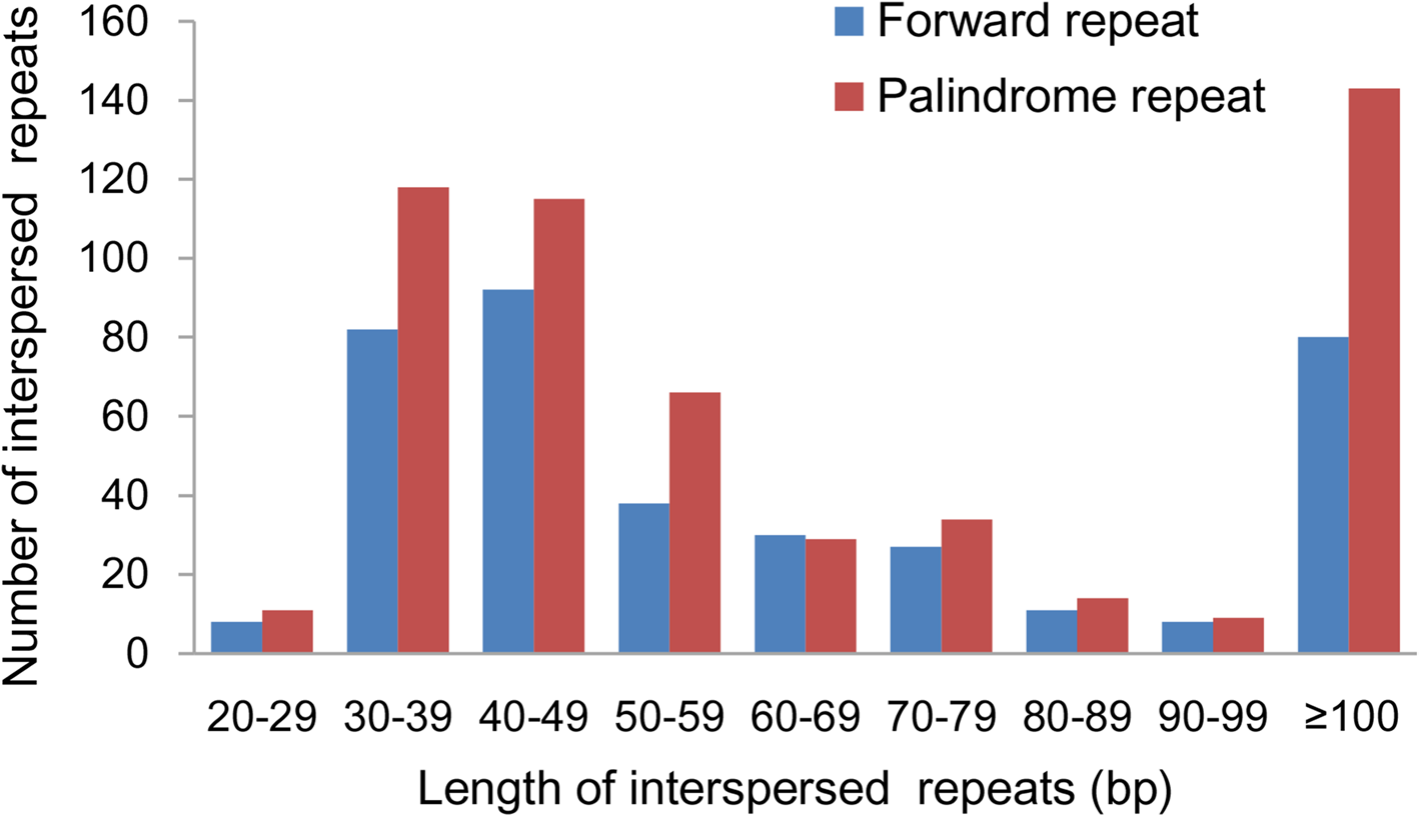
Distribution of lengths of interspersed repeats in the *C. ensifolium* mitogenome

There are three types of repetitive sequences in plants: simple sequence repeats (SSRs), tandem repeats, and dispersed repeats [16]. Figure 4 shows all the types of repetitive sequences found in the *C. ensifolium* mitogenome. The number of SSRs, tandem repeats, and dispersed repeats was 162, 45, and 915, respectively, totaling 1122. Among them, dispersed repeats had the highest number, ranging in length from 27 to 759 bp (data not shown). Of these, 376 were forward repeats, and 539 were palindromic repeats. The longest repeat sequence of the forward type had a length of 759 bp, while the palindromic type had a length of 515 bp. The total length of the dispersed repetitive sequences accounted for 13.78% of the total length of the *C. ensifolium* mitogenome, which equaled 77,244 bp. The abundance of forward repeats was highest in the range of 40–49 bp, while that of palindromic repeats was highest in the range of 30–39 bp (Fig. 5).

SSRs, also known as microsatellite markers, are simple repetitive sequences that are evenly distributed in the genome of eukaryotes. They consist of 1–6 nucleotide tandem repeat fragments. Due to their high variability and the abundance of repeat units between individuals, microsatellite markers have extensive applications [33]. These repeats are relatively evenly distributed across 19 mitochondrial chromosomes, with the distribution quantity of SSRs per chromosome ranging from 5 to 16. The detected SSR sites were classified into five types based on the number of bases: monomer, dimer, trimer, tetramer, and pentamer repeats. Monomer repeats and tetramer repeats were the most abundant types, with both totaling 53. However, tetramer repeats had a greater variety. Together, they constituted 65.43% of the total identified SSRs. Trimer and dimer repeats accounted for 17.90% and 14.81% of the total SSRs, respectively. Pentamer repeats had the lowest number, with only 2 types. Monomer repeats consisted only of A or T bases, without C or G bases (Table 3).

**Table 3:** Types of SSRs detected in the *C. ensifolium* mitogenome (considering sequence complementary)

SSR type	Repeats	Number of SSRs	Total
monomer	A/T	53	53
dimer	AC/GT	2	24
	AG/CT	13	
	AT/AT	9	
trimer	AAC/GTT	1	29
	AAG/CTT	19	
	AAT/ATT	1	
	ACT/AGT	3	
	AGC/CTG	2	
	AGG/CCT	1	
	ATC/ATG	2	
tetramer	AAAC/GTTT	1	53
	AAAG/CTTT	24	
	AACT/AGTT	1	
	AAGC/CTTG	1	
	AAGG/CCTT	1	
	AAGT/ACTT	1	
	AATG/ATTC	4	
	ACAG/CTGT	1	
	ACAT/ATGT	1	
	ACCG/CGGT	1	
	ACGG/CCGT	1	
	ACGT/ACGT	1	
	ACTC/AGTG	2	
	ACTG/AGTC	4	
	AGAT/ATCT	4	
	AGCC/CTGG	1	
	AGCG/CGCT	1	
	AGCT/AGCT	1	
	ATCC/ATGG	1	
	CCGG/CCGG	1	
pentamer	AGAGG/CCTCT	2	3
	ATATC/ATATG	1	
total		162	162

A total of 45 tandem repeats, ranging in length from 9 to 70 bp and with a match degree greater than 68%, were found in the genome. The distribution of tandem repeats was uneven in the *C. ensifolium* mitogenome, with *C. ensifolium chr2* and *C. ensifolium chr7* each having the most (6) tandem repeats, while *C. ensifolium chr13* did not have any (Table S6).

### Phylogenetic analysis

**Fig. 6:**
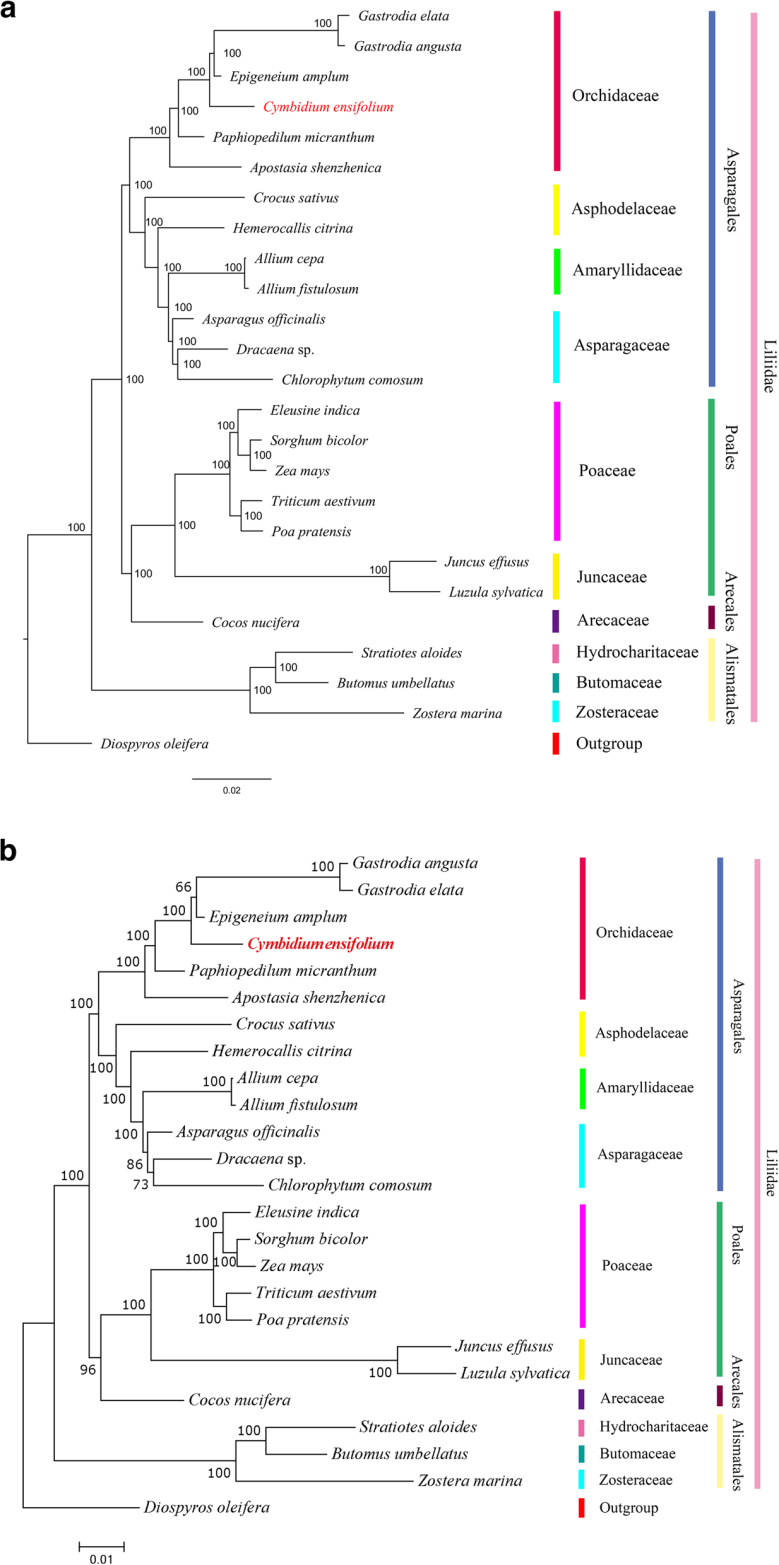
Phylogenetic relationships of *C. ensifolium* with 24 other plant species. **a** Majority-rule consensus tree constructed using the MrBayes method; **b** phylogenetic tree constructed using the maximum-likelihood method. *D. oleifera* served as an outgroup. The bootstrap values are listed at each node. Colors indicate the groups to which the specific species belong

To analyze the evolutionary status of the *C. ensifolium* mitogenome, a phylogenetic analysis was conducted, including 24 other published plant mitogenomes as well as *Diospyros oleifera* Cheng as an outgroup. A phylogenetic tree was created using two different software programs, RAxML and MrBayes (Fig. 6). Both methods yielded the same clustering results, and the branches of the MrBayes majority-rule consensus tree had a high bootstrap support value of 100%. The analysis revealed that all species, including *C. ensifolium*, clustered into four taxa (Asparagales, Poales, Arecales and Alismatales), consistent with the APG IV taxonomic tree [34]. *C. ensifolium*, belonging to the Orchidaceae family, showed the closest genetic relationship to *E. amplum*. This clustering supports the reliability of the mitogenome-based analysis. Based on these relationships, further comparative analysis focused on the Orchidaceae plants.

### Substitution rates of protein-coding genes

**Fig. 7:**
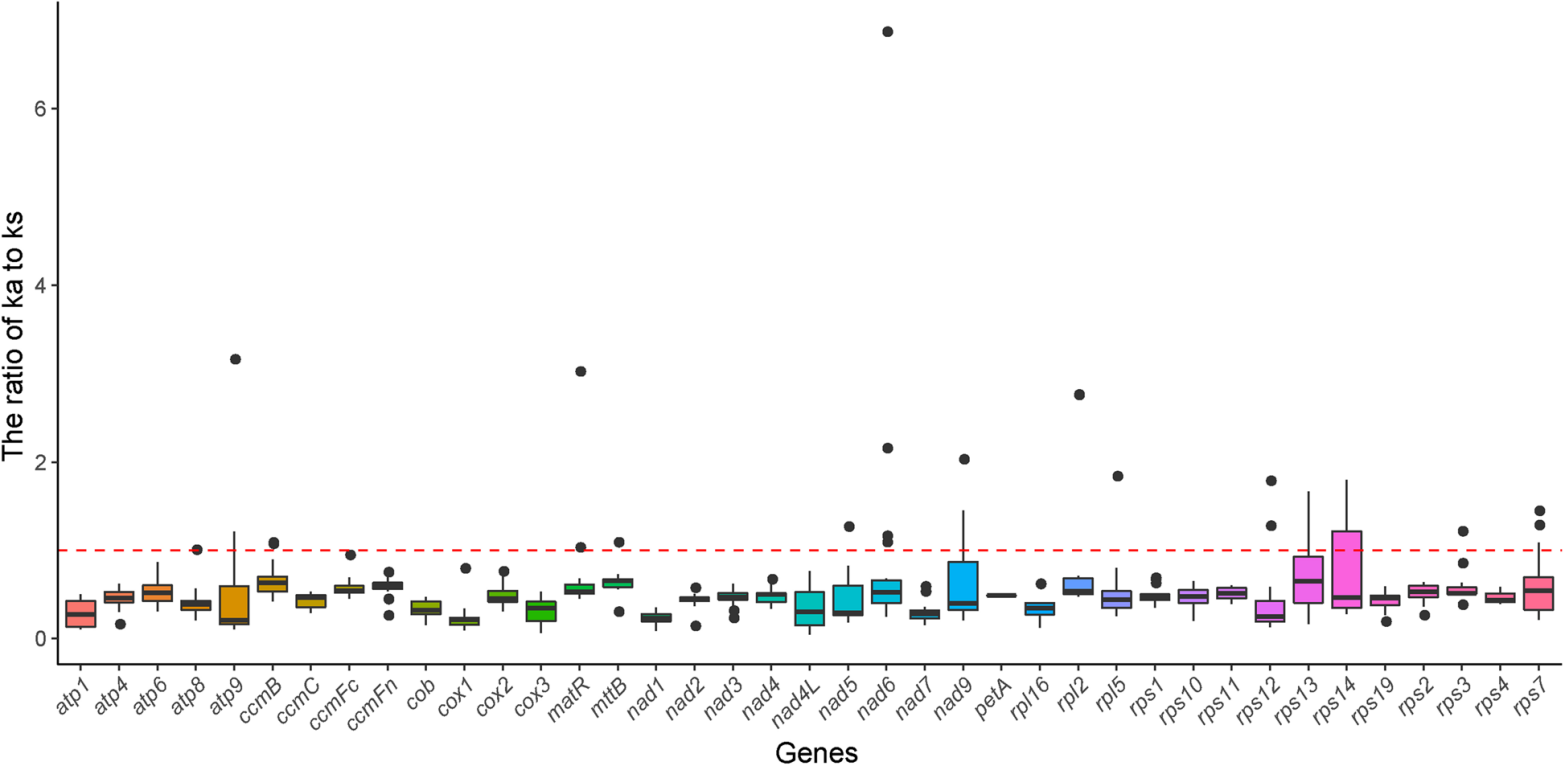
Boxplots of the pairwise Ka/Ks values among all shared mitochondrial genes of the 7 plants primarily in Orchidaceae

Nucleotide variations that do not result in amino acid changes are referred to as synonymous mutations, while those that do cause amino acid changes are called nonsynonymous mutations. It is generally believed that synonymous mutations are not affected by natural selection, while nonsynonymous mutations are influenced by natural selection. Understanding the rates at which synonymous and nonsynonymous mutations occur is meaningful in evolutionary analysis. The commonly used parameters are synonymous mutation frequency (Ks), nonsynonymous mutation frequency (Ka), and the ratio of nonsynonymous to synonymous mutation rates (Ka/Ks). If Ka/Ks > 1, positive selection is inferred. If Ka/Ks = 1, neutral selection is assumed. If Ka/Ks < 1, purifying selection is believed to be involved [35]. The Ka/Ks calculation was performed for the 35 PCGs from the *C. ensifolium* mitogenome compared to the mitogenomes of 6 other plants primarily belonging to Orchidaceae. The results showed that the gene-specific substitution rates, Ka/Ks, ranged from 0.049 for the nad4L gene to 6.868 for the nad6 gene (Fig. 7). The Ka/Ks values of all genes were generally less than 1 in most species, indicating negative selection during evolution. Among them, the *cox1* gene had the smallest average Ka/Ks value (0.228), less than 0.8 in all species, suggesting strong purifying selection and high conservation during the evolutionary process in Orchidaceae plants [36].

### Nucleotide diversity

**Fig. 8:**
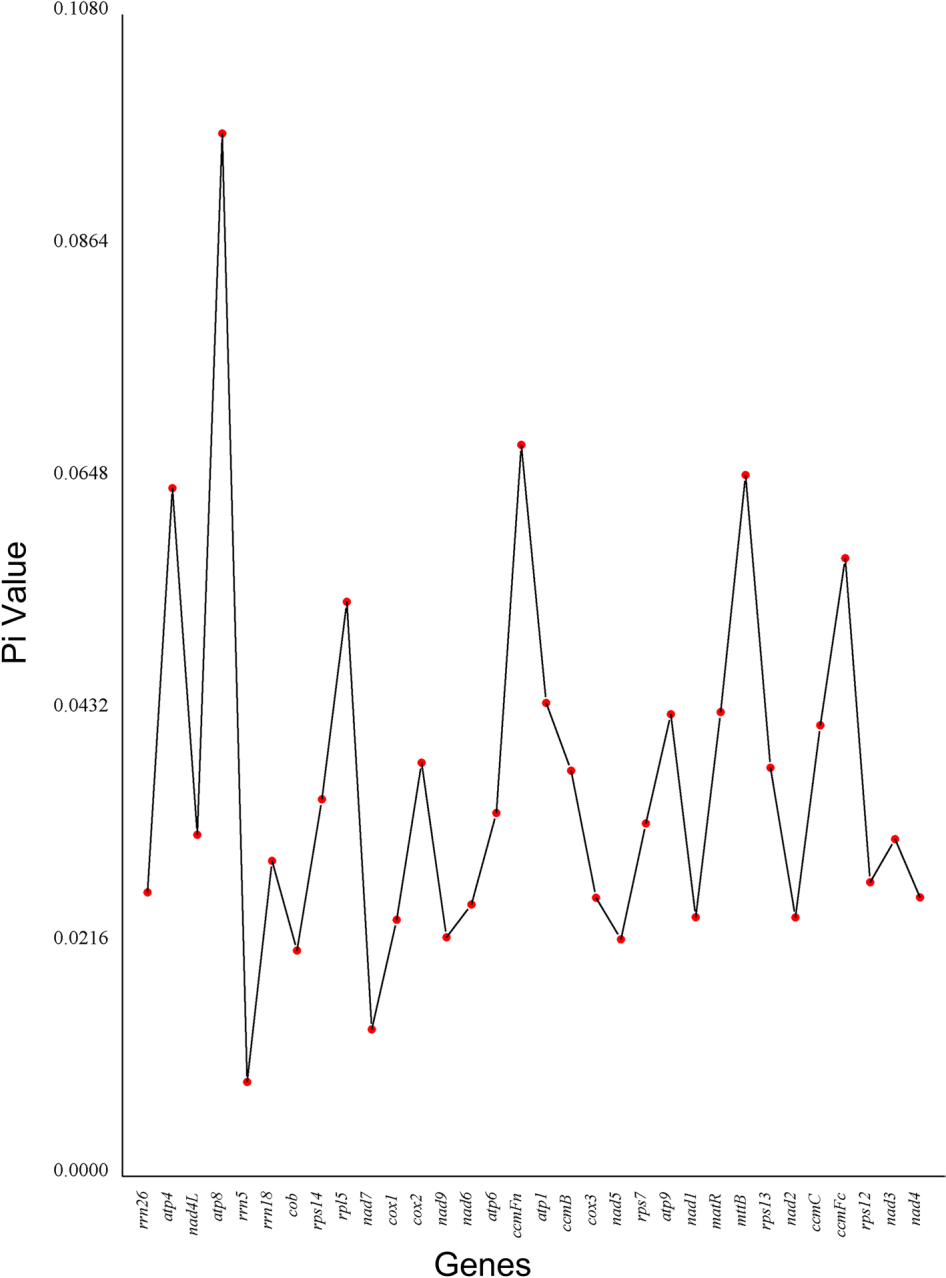
Nucleotide diversity (Pi) among the seven selected plant mitogenomes

Nucleotide diversity (Pi) refers to the probability of nucleotide sequence differences between any two loci in a group of species or a genome. It is an important indicator for evaluating genetic diversity and can also help locate potential molecular marker distribution regions [37]. The nucleotide diversity of the 29 PCGs and 3 rRNA genes among the seven selected plants is shown in Fig. 8. The Pi values of 32 genes ranged from 0.009 to 0.097, with most values being lower than 0.06. Among the PCGs, *atp8* displayed the highest variability (Pi = 0.097), and *ccmFn* (Pi = 0.068), *mttB* (Pi = 0.065), and *atp4* (Pi = 0.064) were also highly variable. On the other hand, *nad7* was the most conserved PCG (Pi = 0.014). In summary, the nucleotide diversity of the PCGs was highly variable. Furthermore, all three rRNA genes were conserved, with values of 0.009 for *rrn5*, 0.026 for *rrn26*, and 0.029 for *rrn18*.

### Analysis of homologous fragments between mitochondria and chloroplasts

**Fig. 9:**
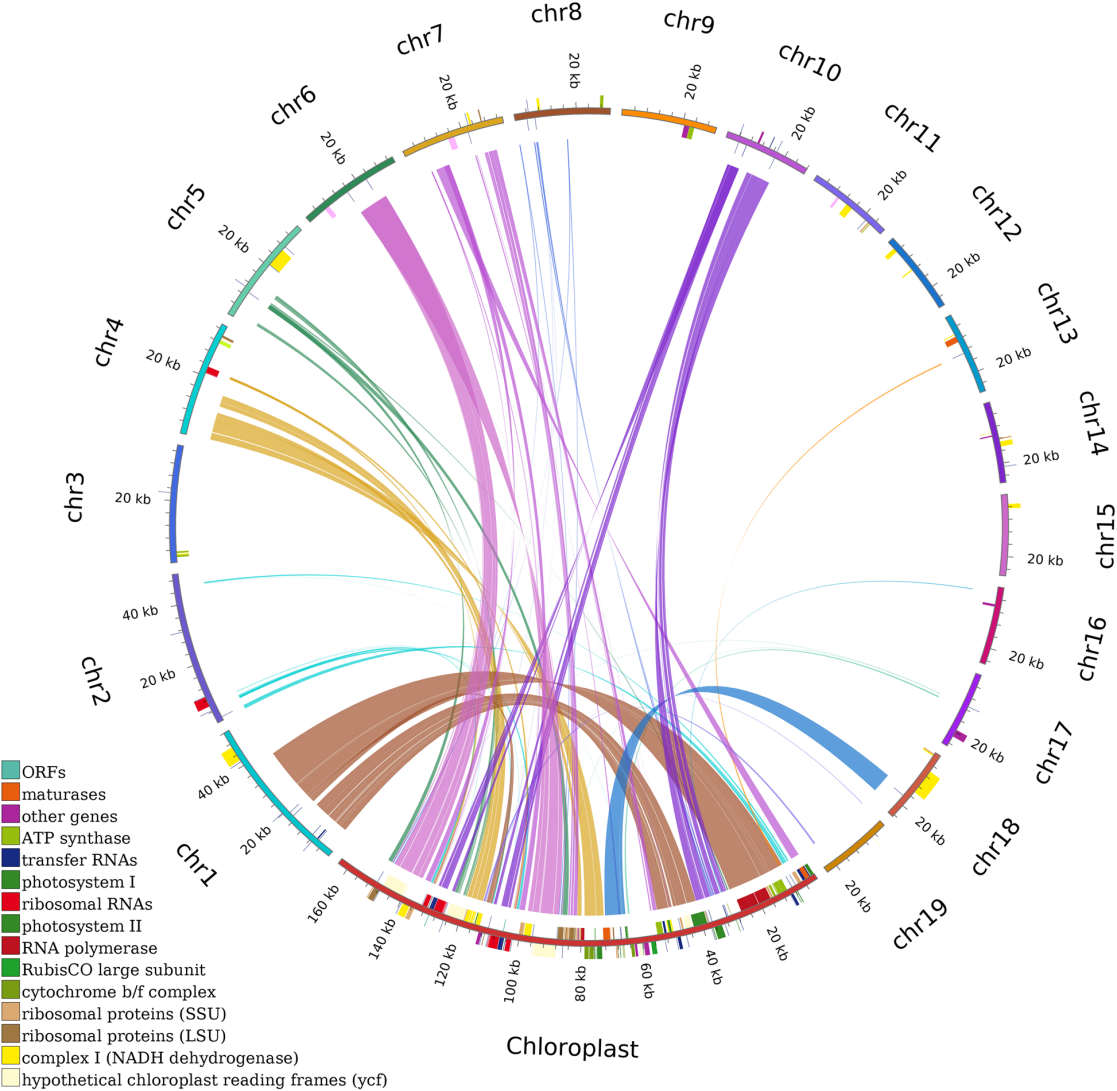
Distribution of homologous fragments between mitochondria and chloroplasts in *C. ensifolium*

Homologous fragments between the *C. ensifolium* mitogenome and chloroplast genome were detected and analyzed (Fig. 9). Out of the 19 chromosomes in the *C. ensifolium* mitogenome, 13 contained sequences derived from the plastome, resulting in a total of 117 homologous fragments. These fragments varied in length from 37 to 11,412 bp, with a combined length of 38,163 bp. These fragments accounted for 6.81% of the mitogenome and 79.39% of the *C. ensifolium* plastome (Table S7). The homologous fragments showed a high similarity to their corresponding conspecific PCGs, with 48 chloroplast PCGs completely located within the homologous regions, showing a percentage of identical matches ranging from 86.2% to 98.4%. Furthermore, 23 tRNA genes exhibited a percentage of identical matches ranging from 76.7% to 98.1%. Various partial genes and intergenic spacer regions were also identified (Table S7). The chromosome with the largest plastome-derived sequence, *C. ensifolium chr1*, had a length of 34,674 bp out of a total length of 48,212 bp, accounting for 71.92% of the *C. ensifolium chr1* mitogenome. Most of the plastome-derived sequences showed a high degree of similarity to their corresponding conspecific plastome sequences, ranging from 74.3% to 100.0%.

### Analysis of homologous fragments between the mitochondrial and nuclear genomes

**Fig. 10:**
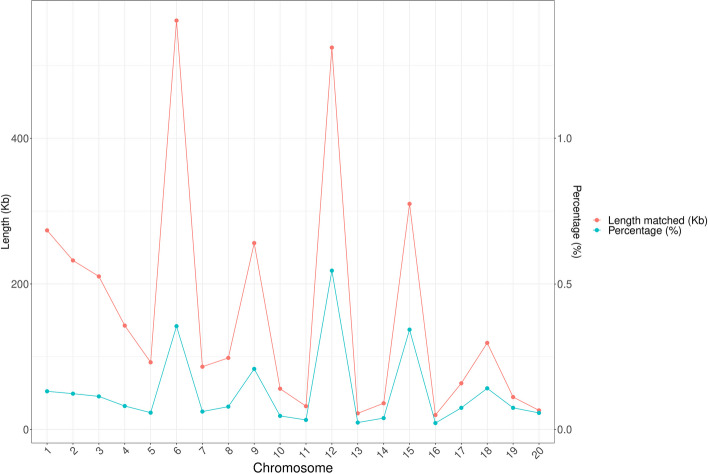
The length and proportion of homologous fragments between the nuclear and mitochondrial genomes for each nuclear genome chromosome in *C. ensifolium*

Homologous fragments between the *C. ensifolium* mitogenome and nuclear genome were detected and analysed (Table S8). These homologous fragments were found across all 19 mitogenome chromosomes and 20 nuclear chromosomes in *C. ensifolium*. The lengths of homologous fragments on the 20 nuclear chromosomes ranged from 19.94 kb to 561.86 kb, accounting for 2.23% to 54.57% of the respective chromosome length (Fig. 10). The longest homologous fragment was 561.86 kb on nuclear chromosome 6, accounting for 35.50% of the total length, followed by 524.74 kb on nuclear chromosome 12, accounting for 54.57%. The shortest homologous fragment was 19.94 kb on nuclear chromosome 16, representing 2.23%. These homologous fragments contained numerous long homologous sequences (> 500 bp), with significant numbers (1728 and 1266) of segments ranging from 500–1000 bp and 1000–2000 bp, respectively. Additionally, there were 114 segments with homologous sequences exceeding 4000 bp (data not shown). The abundant homologous fragments in the nuclear genome contained multiple genes from the mitochondria, including 5 protein-coding genes (*atp8*, *ccmFn*, *mttB*, *nad9*, and *rps13*), 2 rRNA genes (*rrn18* and *rrn5*), and 11 tRNA genes (*trnC-GCA*, *trnF-GAA*, *trnL-TAG*, *trnM-CAT*, *trnQ-TTG*, *trnR-ACG*, *trnS-AGA*, *trnS-GCT*, *trnS-GGA*, *trnW-CCA*, and *trnY-GTA*) completely within the homologous fragments. Almost all coding sequences could be found in the homologous fragments, although some were incomplete and showed low homology. Except for *ccmFc*, no homologous sequences were found in the homologous fragments.

### Synteny analysis of mitochondrial sequences

**Fig. 11:**
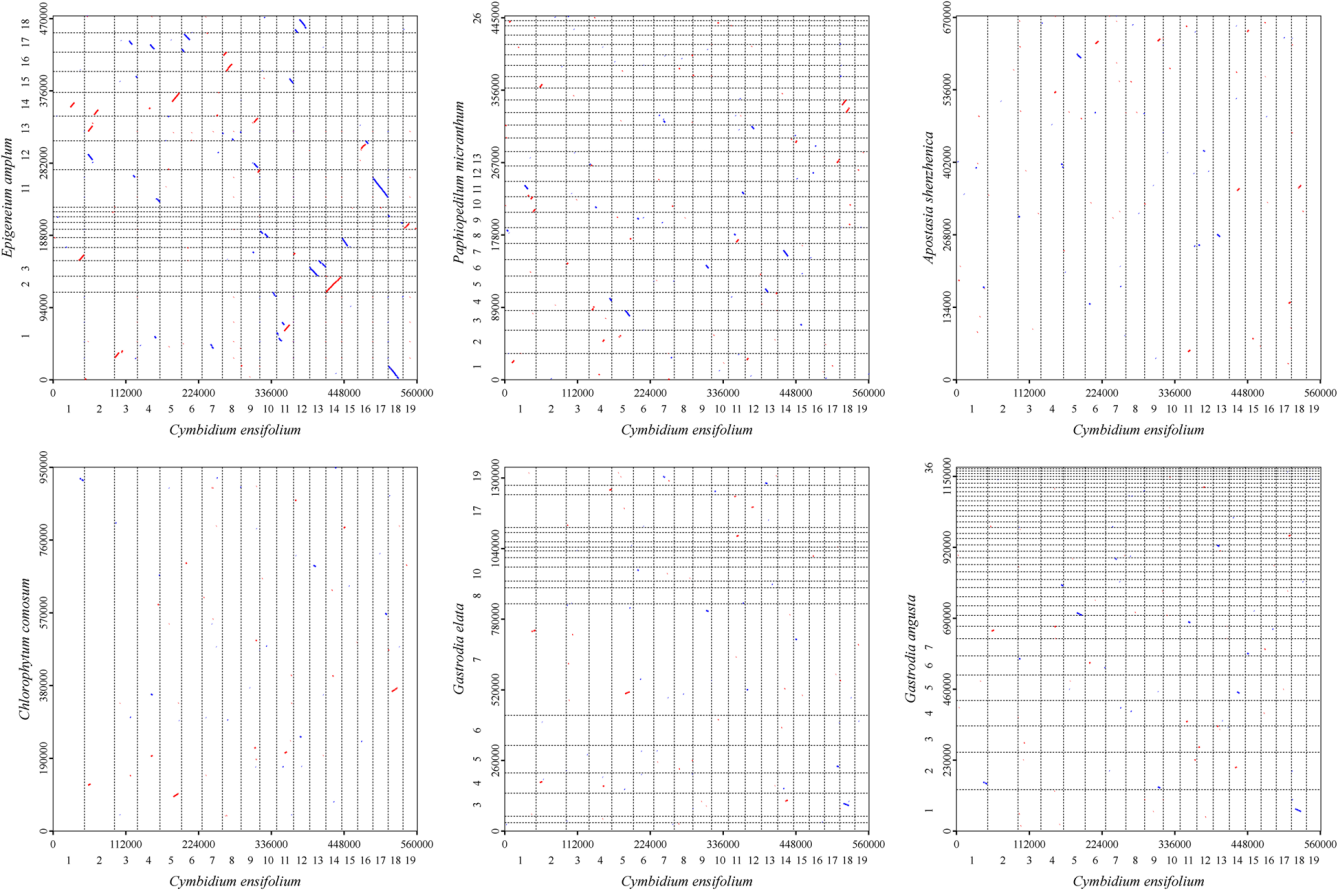
Dot-plot graphs illustrating syntenic sequences between mitogenomes primarily in Orchidaceae plants compared to *C. ensifolium* as the reference. The red line in the box represents forward comparison, while the blue line represents reverse complementary comparison

**Fig. 12:**
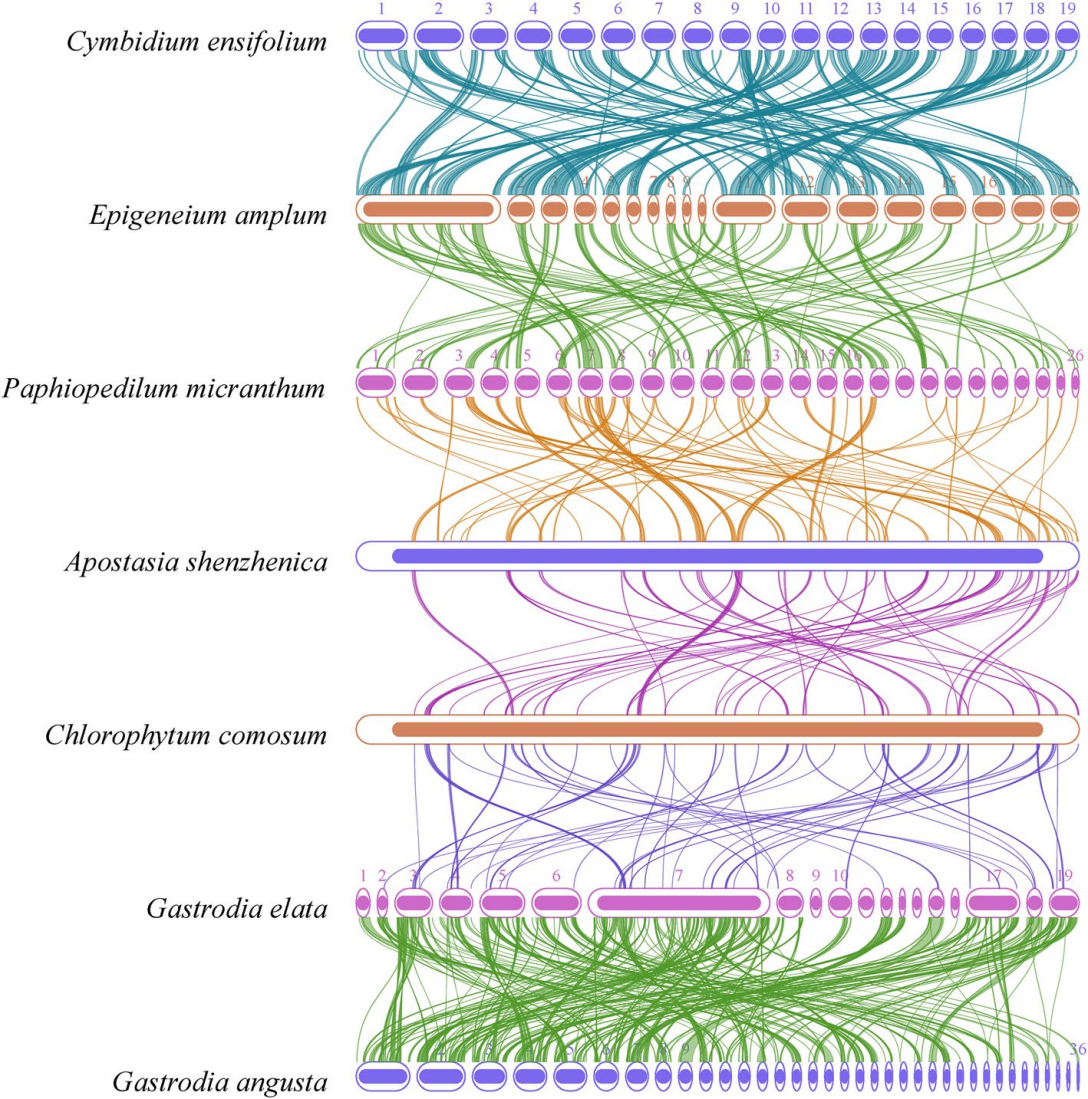
Collinearity plots of the mitogenomes of *C. ensifolium* and the other Orchidaceae plants. The boxes in each row indicate the mitogenomes, and the connecting lines in the middle indicate homologous regions

The synteny analysis of mitochondrial sequences, as shown in dot-plot analysis (Fig. 11), revealed the longest syntenic sequences with the highest similarity between *C. ensifolium* and *E. amplum*, indicating a close genetic relationship between these species. Pairwise synteny analysis indicated the presence of numerous homologous colinear blocks between individual mitogenomes, although they were not arranged in the same order. This suggests that the structure of the seven plant mitogenomes is highly nonconserved, with widespread rearrangement events. The largest number of homologous sequences was found between the *G. elata* and *G. angusta* mitogenomes, two plants that belong to the same genus. Additionally, the number of homologous sequences between the *C. ensifolium* and *E. amplum* mitogenomes was much higher than that between the other pairs (Fig. 12).

**Fig. 13:**
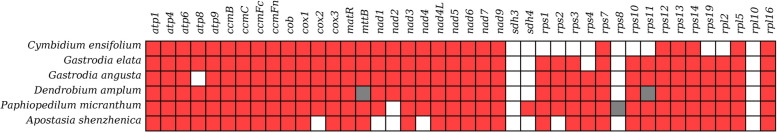
Comparison of mitochondrial protein-coding genes among 6 species of the Orchidaceae family

Furthermore, the conservation analysis of PCGs in six species of the Orchidaceae family revealed significant differences in the types and quantities of PCGs among the species. For example, *P. micranthum* had the most PCGs (39), while *C. ensifolium* had the fewest (30), mainly due to the significant loss of ribosomal protein-coding genes. In *A. shenzhenica*, multiple NADH dehydrogenase-encoding genes (*nad1*, *nad2*, and *nad4*) were lost, and the copy numbers of these genes were significantly reduced (data not shown). Additionally, several orchid species commonly lacked *sdh3, sdh4*, *rps8*, and *rpl10*, with only *P. micranthum* containing *sdh4* and the pseudogene for *rps8* (Fig. 13).

### Comparison of mitogenome size, structure, gene content and GC content with those of plants containing a multichromosomal mitogenome

The mitogenome of *C. ensifolium* is unique compared to that of most plants, as it is assembled into 19 circular chromosomes, whereas most plants have a single circular chromosome structure [31]. To further understand its characteristics, the mitogenome of *C. ensifolium* was compared to those of 47 other plants with multichromosomal mitogenome structures (Table S9) [14–16, 21, 22, 24, 27, 28, 38–67]. The number of submitochondrial chromosomes varied from 2 to 130 among different species ([40], Table S9). The genome sizes of the selected plants also varied greatly, ranging from 65,874 bp (*Viscum scurruloideum* Barlow) to approximately 11.7 Mb (*Larix sibirica* Ledeb.). The size of the subgenome within the mitogenome also varied greatly, with the smallest subgenome being only 1,588 bp (*Silene vulgaris* Garcke) and the largest reaching approximately 5360 kb (*Picea glauca* Voss). The GC content of the plants ranged from 40.8% to 51.2%, with the fern-like plant *Psilotum nudum* (L.) P. Beauv. having the highest GC content. Common genes, such as *rrn5*, *rrn18*, and *rrn26*, were found in most plants, but the total number of genes varied significantly, ranging from 31 in *E. amplum* to 143 in *P. glauca*. The mitochondrial genome structures of the selected plants were also diverse, with some containing two circular subgenomes, some having linear subgenomes, and others having a combination of circular and linear subgenomes (NCBI database, [14, 59, 68]).

## Discussion

### General features of the* C. ensifolium* mitogenome

Mitochondria are organelles in eukaryotic cells that have their own genetic system and are responsible for energy production through RNA expression and protein synthesis [17]. In many plant species, mitogenomes are typically visualized as a single circular molecule without any isoforms [24, 31]. However, electron micrographs have shown that the mitogenome of *Chenopodium album* L. contains a subgenome consisting of a circular molecule with a linear tail [68]. With advancements in sequencing technology, particularly third-generation sequencing, more mitogenomes are being successfully assembled, revealing complex structures. For example, the mitogenome of *P. micranthum* consists of 26 circular subgenomes [16]. The increasing availability of high-quality genome sequencing data in the NCBI database allows for more in-depth research, contributing to progress in genomics research and saving resources [13, 14, 69, 70].

By utilizing the raw genome sequences obtained from BioProject/GSA under the accession codes PRJCA005355/CRA004327, we successfully assembled the mitogenome of *C. ensifolium*. The mitogenome consisted of 19 circular chromosomes with a total length of 560,647 base pairs. Compared to other Orchidaceae plants in the NCBI database (NCBI database, [14–16]), such as *G. angusta* (1181 kb with 36 circular subgenomes), *C. lancifolium* (704 kb with 23 circular subgenomes), *P. micranthum* (447 kb with 26 circular subgenomes), and *E. amplum* (461 kb with 18 linear subgenomes), the genome size and subgenome quantity of *C. ensifolium* were moderate. The *C. ensifolium* mitogenome consisted of 35 PCGs, which was similar to the number in *C. lancifolium* (38) and *C. macrorhizon* (38). However, the gene content in the *C. ensifolium* mitogenome differed significantly from that in the mitogenomes of the other two *Cymbidium* plants (Table 4). Notably, 8 ribosomal proteins were lost in the *C. ensifolium* mitogenome. Although the loss of ribosomal proteins has been observed in *Dendrobium* mitogenomes [71], the extent of ribosomal protein loss in the *C. ensifolium* mitogenome was remarkable. This indicates that the mitogenomes of *Cymbidium* plants underwent rapid structural evolution.

**Table 4:** The protein-coding genes in the *C. ensifolium*, *C. lancifolium* and *C. macrorhizon* mitogenomes

Group of protein-coding genes	*C. ensifolium*	*C. lancifolium*	*C. macrorhizon*
ATP synthase	*atp1*	*atp1*	*atp1*
	*atp4*	*atp4*	*atp4*
	*atp6*	*atp6*	*atp6*
	*atp8*	*atp8*	*atp8*
	*atp9*	*atp9*	*atp9*
Cytochrome c biogenesis	*ccmB*	*ccmB*	*ccmB*
	*ccmC*	*ccmC*	*ccmC*
	*ccmFc*	*ccmFc*	*ccmFc*
	*ccmFn*	*ccmFn*	*ccmFn*
Ubiquinol cytochrome c reductase	*cob*	*cob*	*cob*
Cytochrome c oxidase	*cox1*	*cox1*	*cox1*
	*cox2*	*cox2*	*cox2*
	*cox3*	*cox3*	*cox3*
Maturases	*matR*	*matR*	*matR*
Transport membrane protein	*mttB*	*mttB*	*mttB*
NADH dehydrogenase	*nad1* (3)	*nad1*	*nad1*
	*nad2* (2)	*nad2*	*nad2*
	*nad3*	*nad3*	*nad3*
	*nad4*	*nad4*	*nad4*
	*nad4L*	*nad4L*	*nad4L*
	*nad5* (3)	*nad5*	*nad5*
	*nad6*	*nad6*	*nad6*
	*nad7*	*nad7*	*nad7*
	*nad9*	*nad9*	*nad9*
Ribosomal proteins (LSU)	*rpl16*	*rpl16*	*rpl16*
	×	*rpl2*	*rpl2*
	*rpl5*	*rpl5*	*rpl5*
Ribosomal proteins (SSU)	×	*rps1*	*rps1*
	×	*rps10*	*rps10*
	×	*rps11*	*rps11*
	*rps12*	*rps12*	*rps12*
	*rps13*	*rps13*	*rps13*
	*rps14*	*rps14*	*rps14*
	×	*rps19*	*rps19*
	×	*rps2*	*rps2*
	×	*rps3*	*rps3*
	×	*rps4*	*rps4*
	*rps7*	*rps7*	*rps7*
Total number of PCGs	35	38	38

The numbers after gene names indicate the number of copies; “x” indicates gene loss

RNA editing, which mainly occurs in coding transcripts of plant organelles, is thought to play a role in regulating gene expression [72]. In our study, we predicted 530 RNA editing sites in the *C. ensifolium* mitogenome, which is similar to the number reported in previous studies on *Hypopitys monotropa* Crantz (545) [54], *B. chinense* (517) [73], and *Ilex metabaptista* Loes. ex Diels (543) [31]. The number of RNA editing sites in PCGs varied greatly among genes and species. In our study, cytochrome c biogenesis and NADH dehydrogenase genes had the most editing sites, resembling the patterns observed in *I. metabaptista* [31] and *B. chinense* [73]. In contrast, in *Ipomoea batatas* (L.) Lam., the ribosomal protein genes *rpl16* (49) and *rps3* (54) contained the largest number of editing sites [74]. Additionally, all the RNA editing sites in the *C. ensifolium* mitogenome were located at the first two codon positions and exhibited a C-T editing type, which is commonly observed in plants [31].

Analysis of codon usage bias is important for studying species origin and genetic differentiation [32]. In the *C. ensifolium* mitogenome, all PCGs used the typical ATG start codon. This differed from the start codon usage observed in *I. metabaptista*, where *nad4L* and *rps10* used ACG and *rps4* used TTG, likely as a result of RNA editing [75]. We found that almost all of the RSCU values for the NNT and NNA codons were higher than 1.0, with the exceptions of the values for Ile (AUA, 0.773), Leu (CUA, 0.832), and Ala (GCA, 0.939), which was similar to observations in *Acer truncatum* Bunge [75]. Codon usage in the *C. ensifolium* mitogenome was generally biased toward A or T(U) at the third codon position, which is a common pattern in the mitogenomes of land plant species [76].

Repeats are commonly present in plant mitochondrial genomes. They can induce repeat-mediated recombination, be associated with horizontal gene transfer, and provide SSR markers for species identification [57, 77]. In the *C. ensifolium* mitogenomes, 1122 repeats were identified, including 162 SSRs, 45 tandem repeats, and 915 dispersed repeats. The abundance of repetitive sequences suggests that intermolecular recombination is frequent in the *C. ensifolium* mitogenome [31]. Interestingly, all identified monomer SSRs in the *C. ensifolium* mitogenome consisted of A and T bases rather than C and T bases. This could be due to the lower energy required to break the bonds between A and T than to break GC bonds, as observed in *Bupleurum chinense* DC. [73].

### Phylogenetic inference

Plant mitogenome sequences are known to evolve slowly, with an overall ratio of relative synonymous substitution rates among mitochondrial, chloroplast, and nuclear genes in angiosperms of 1:3:16 [78]. Therefore, phylogenetic analysis is typically based on nuclear genes and genome data or chloroplast genes and genome data [37, 79]. With the increase in mitochondrial genome sequencing data, significant differences in mitochondrial genomes, including size and diverse structural abundance, have been observed. Mitogenomes have become a valuable tool for studies in taxonomy, phylogenetics, evolution, population genetics, and comparative genomics [49, 71]. In this study, a phylogenetic analysis of the *C. ensifolium* mitogenome and 24 published plant mitogenomes was conducted. Both construction methods yielded the same clustering result, indicating the consistency of traditional and molecular taxonomies. Previous research on the *I. metabaptista* mitogenome and the mitogenomes of 29 selected plant species also demonstrated the accuracy of constructing trees based on mitochondrial genomes, highlighting the potential of using mitogenome information in plant phylogenetic studies [31]. *C. ensifolium*, an ornamental flower with notable morphological diversity and numerous subspecies, shows differences in economic value among subspecies. Methods for distinguishing subspecies mainly rely on morphological classification, SSRs, RAPD markers, etc. [80–82]. However, intraspecific variation in mitochondrial genome sequence, structure, and gene content has been observed in other species [51, 83], providing a new method for distinguishing *C. ensifolium* subspecies. Nonetheless, more mitogenomes of *C. ensifolium* subspecies and other orchids need to be sequenced to further explore subspecies identification, phylogenetic relationships, and evolutionary biology within this large family.

### Intracellular gene transfer in the mitogenome of *C. ensifolium*

Previous studies have revealed that DNA transfer events between different genomes (mitochondrial, nuclear, and chloroplast) exist, with the most significant transfer direction in angiosperms being from organellar genomes into the nuclear genome. This is followed by transfer from nuclear and plastid genomes into the mitogenome [16, 22, 84, 85]. Analysis and statistics were conducted on plastid-derived DNA sequences in the mitogenome of *C. ensifolium*. A total of 38,163 sequences, accounting for 6.81% of the mitogenome, showed homology to the conspecific chloroplast sequence. This percentage is similar to those found in *C. lancifolium* and *C. macrorhizon* mitogenomes (5% ~ 6%) [15], lower than that in *P. micranthum* (10.34%) [16], and higher than those in *B. chinense* (2.56%) and *A. truncatum* (2.36%) [73, 75]. Additionally, the homologous fragments exhibited high similarity to their conspecific PCGs, with 48 chloroplast PCGs located completely within the homologous regions, showing a percentage of identical matches ranging from 86.2% to 98.4%. Furthermore, 23 types of transfer RNA genes between the *C. ensifolium* mitogenome and the conspecific chloroplast genome showed a percentage of identical matches ranging from 76.7% to 98.1%, which is common in angiosperms [15, 75].

With an increasing number of fully sequenced mitochondrial genomes, more instances of mitochondrial-to-nuclear gene transfer are being discovered [86]. In this study, 5 PCGs (*atp8*, *ccmFn*, *mttb*, *nad9*, and *rps13*), 2 rRNA genes, and 11 tRNA genes in *C. ensifolium* were found to be completely located in homologous sequences between the mitochondrial genome and the nuclear genome, covering 7 out of 11 PCG groups. Widespread gene exchange between the mitochondrial and nuclear genomes in *C. ensifolium* was observed in terms of the length and distribution of homologous sequences. Studies have shown that DNA transfer from mitochondria to the nucleus can lead to a large number of mutation sites in the nuclear genome, increasing its diversity and potentially explaining the presence of hundreds of subspecies in *C. ensifolium* [87]. The genes transferred from the mitochondrial genome to the nuclear genome may vary among species, and there is no clear unified transfer pattern, indicating that intracellular gene transfer is, to some extent, a random and independent event [22, 86, 88–92]. However, confirmation of this hypothesis is limited by the small number of species analysed to date for mitochondrial genome-to-nuclear genome comparisons. With more sequencing data being published and analysed, it may be possible to determine the level and mechanisms of intracellular gene transfer on the basis of larger datasets.

In addition to intracellular gene transfer, the growth cycle of orchid plants is closely related to fungi, increasing the probability of horizontal gene transfer between the mitogenomes of fungi and the ancestors of orchids. Previous research has shown that the ancestor of orchids acquired an ~ 270 bp fungal mitogenomic region containing three transfer RNA genes [25], and it is expected that new evidence will be discovered with the improvement of mitogenome databases.

### Mitogenome comparison in Liliidae and plants with multichromosomal mitogenome structures

An increasing amount of mitogenome sequence information can be found in online databases [15, 36, 48, 61]. To gain a better understanding of its structure and organization, we compared the mitogenome of *C. ensifolium* to those of Liliidae and plants with multichromosomal structures. The selected plants displayed highly nonconserved structures and genome sizes, suggesting that there have been numerous gene loss, gene gain, and rearrangement events between different mitogenomes [20, 21, 55, 71].

The Ka/Ks value can be used to determine whether a specific protein-coding gene has been under selection pressure during evolution [73]. Ka/Ks ratios for all common genes in the Orchidaceae mitogenomes were less than one, indicating purifying selection, which are similar to the ratios observed in Isochrysidales mitogenomes [76]. However, Ka/Ks ratios greater than one have been reported for some mitochondrial genes. For example, the Ka/Ks value of PCGs such as *ccmB* and *nad4* was found to be greater than that in the mitogenomes of *B. chinense* and *Bupleurum falcalum* L. [73]. Among the selected asterids, the *ccmB* gene also had a Ka/Ks ratio greater than one [31]. These results suggest that these genes underwent positive selection during evolution and are important for further studies on gene selection and evolution within their respective groups [75].

Genome size, structure, gene content, and GC content are important factors in assessing species [15]. We compared these characteristics of the *C. ensifolium* mitogenome with those of 47 other plants with multichromosomal structures (Table S9). While genome size, structure, and gene content differed greatly, the GC content was relatively consistent among the selected plants. This suggests that GC content is highly conserved during the evolutionary process of higher plants [16]. To better understand the reasons for these significant differences, more detailed comparisons will need to be conducted in future studies.

The mitochondrial gene content varies greatly among eukaryotes [85, 93]. Our analysis revealed that orchids exhibit a particularly notable pattern. Comparative analysis revealed that among the 11 PCG groups, only the cytochrome c biogenesis gene was conserved, while the remaining genes showed varying degrees of divergence. The conservation of PCGs in orchids is much lower than that in cereal crops [24]. Keith et al. conducted a protein-coding gene alignment analysis of 280 angiosperms, identifying 24 relatively conserved genes with only one instance of gene loss each for *atp8* and *cox2*. However, the orchid species *A. shenzhennica* has lost three NADH dehydrogenase genes (*nad1*, *nad2*, and *nad4*), a significant difference compared to other orchids [94]. Notably, among the six selected orchid species, only *A. shenzhennica* has a single-circular mitochondrial genome structure; the others have multiple circular structures. The underlying cause of these differences, possibly due to different evolutionary strategies, warrants further investigation. The loss of the *sdh3*, *sdh4* and *rpl10* genes is common in the Orchidaceae family, similar to the pattern in most monocots [24]. Conversely, the *C. ensifolium* mitogenome has lost eight ribosomal proteins, a rare phenomenon in orchids but more common in the genus *Silene* [49]. With the increasing availability of orchid genome sequencing data, future research may shed light on the reasons for these genetic patterns.

## Methods

### Raw data acquisition and filtering

The raw data used for assembling the mitochondrial genome of *C. ensifolium* were obtained from the National Genomics Data Center (NGDC). The assembled and annotated whole-genome data of *C. ensifolium* have been deposited in BioProject/GWH under accession codes PRJCA005355/GWHBCII00000000 [70]. According to the article, the *C. ensifolium* used in the study was an adult wild plant found in the Gushan Scenic Area, Fuzhou, Fujian Province [70].

Sequencing was performed using the Illumina HiSeq X-Ten system, and a 20-kb insert library was constructed based on the PacBio RSII protocol for PacBio sequencing [70]. To obtain a high-quality *C. ensifolium* mitogenome, the original data were filtered, and high-quality reads were obtained using fastp (v0.20.0, https://github.com/OpenGene/fastp) software. The third-generation sequencing data were then filtered using Filtlong (v0.2.1, https://link.zhihu.com/?target=https%3A//github.com/rrwick/Filtlong) software and quantified using Perl scripts.

### Mitogenome assembly and annotation

Plant mitochondrial genes, including coding sequences and ribosomal RNA, are highly conserved. Taking advantage of this feature, Minimap2 (v2.1) comparison software was used to compare the original long-read sequencing data with the reference gene sequence data of the plant mitochondrial core genes [95]. Sequences with similar fragments longer than 50 bp were selected as candidate sequences. The candidate sequences with more aligned genes and higher alignment quality were selected as the seed sequence. The original long-read sequencing data were then compared to the seed sequence, and sequences with a minimum overlap of 1 kb and at least 70% similarity were added to the seed sequence. This process was iteratively repeated to obtain all long-read sequencing data of the mitochondrial genome. Canu (v0.4.8) assembly software was used to correct the obtained long-read sequencing data, and Bowtie2 (v2.3.5.1) was used to align the short-read sequencing data to the corrected sequence [96, 97]. Unicycler (v0.4.8) software was used to concatenate the short-read sequencing data and the corrected long-read sequencing data with default parameters to obtain the final circular mitochondrial genome of *C. ensifolium* [98].

The annotation of the mitochondrial genome structure was performed in several steps. PCGs and rRNA were aligned to published plant mitochondrial sequences using BLAST and adjusted manually for related species (Table S10). tRNA was annotated using tRNAscan-SE (http://lowelab.ucsc.edu/tRNAscan-SE/), and ORFs were annotated using Open Reading Frame Finder (http://www.ncbi.nlm.nih.gov/gorf/gorf.html) [99]. Sequences with a length shorter than 102 bp and sequences overlapping with known genes were excluded. Alignments greater than 300 bp in length were annotated against the NR library. The above results were manually checked and corrected for more accurate annotation. The mitochondrial genome was then visualized using OGDRAW (v1.3.1, https://chlorobox.mpimp-golm.mpg.de/OGDraw.html).

### RNA editing analysis

The editing sites in the mitochondrial RNA of *C. ensifolium* were identified using the mitochondrial gene-encoded proteins of plants as reference proteins. The Plant Predictive RNA Editor (PREP) suite (http://prep.unl.edu/) was used for the analysis [100].

### Codon usage analysis

RSCU was analyzed to understand the combined effect of natural selection, mutation, and genetic drift in determining codon usage. A self-encoded Perl script was used to analyze the codon composition of the *C. ensifolium* mitogenome. The analysis included screening for unique CDSs, determining the number of codons per gene, calculating the GC content (GC1, GC2, GC3, and GC all), calculating the effective number of codons (Nc), and analyzing the RSCU of synonymous codons.

### Analysis of repeat sequences

Three types of repeats (simple sequence, tandem, and dispersed) were detected in the *C. ensifolium* mitochondrial genome. The MIcroSAtellite (MISA) identification tool Perl script was used to identify simple sequence repeats (v1.0, parameter: 1–10 2–5 3–4 4–3 5–3 6–3) [101]. Tandem repeats (> 6 bp repeat units) were detected using Tandem Repeats Finder software (v4.09, http://tandem.bu.edu/trf/trf.submit.options.html) (trf409.linux64, parameters: 2 7 7 80 10 50 2000 –f -d -m) with default parameters [102]. Dispersed repeats were detected using BLASTn (v2.10.1, parameters: -word size 7, evalue 1e-5, remove redundancy, remove tandem duplication) with specific parameters. The repeats were visualized using Circos software (v0.69–5, http://circos.ca/software/download/).

### Construction of the phylogenetic tree

To determine the phylogenetic position of *C. ensifolium*, we downloaded 24 plant mitogenomes (Table S11) from the NCBI Organelle Genome Resources database (http://www.ncbi.nlm.nih.gov/genome/organelle/). The shared conserved mitochondrial gene CDSs of 25 species from different families were aligned using MAFFT (v7.427, –auto mode) software [103]. We used two methods to construct the phylogenetic tree. The maximum likelihood (ML) phylogenetic tree was generated using RAxML (v8.2.10, https://cme.h-its.org/exelixis/software.html) with 1000 bootstrap replications, using the GTRGAMMA model for estimation [104]. The optimal nucleotide substitution model was determined using jModelTest (v2.1.10, https://github.com/ddarriba/jmodeltest2), and the Bayesian inference (BI) phylogenetic tree was constructed using MrBayes (v3.2.7a, http://nbisweden.github.io/MrBayes/) with parameters based on the results from jModelTest v2.1.10. We designated *D. oleifera* as an outgroup.

The six selected plants, primarily belonging to Orchidaceae, namely, *G. elata* (MF070084.1-MF070102.1), *G. angusta* (MH591794.1-MH591823.1), *E. amplum* (MH591879.1-MH591896.1), *P. micranthum* (OP465200.1-OP465225.1), *A. shenzhenica* (OQ645347.1), and *Chlorophytum comosum* (Thunb.) Jacques (MW411187.1), were used for analysis of synonymous and nonsynonymous substitution ratios (Ka/Ks), nucleotide diversity (Pi), and synteny. Five Orchidaceae species were also subjected to PCG conservation analysis.

### Analysis of Ka/Ks values

To investigate natural selection pressures during the evolution of the Orchidaceae, we selected six plants. We aligned the shared PCGs using MAFFT v7 [103] and calculated the ratios of nonsynonymous (Ka) to synonymous (Ks) substitutions (Ka/Ks) using KaKs_Calculator v 2.0 [105].

### Analysis of nucleotide diversity

We globally compared homologous gene sequences of different species using MAFFT (v7.427, –auto mode) software [103] and calculated the Pi value of each gene using DnaSP v5 [106].

### Comparative analysis of mitochondrial genomes

We performed genome alignment between the *C. ensifolium* mitogenome and six mitogenomes of selected plants using nucmer (4.0.0beta2) [107] software with the maxmatch parameter to generate dot plots. We used BLASTN (v2.10.1 +) software to draw collinearity plots of *C. ensifolium* and selected plants, with a word size set to 7, an e-value set to 1E-5, and screening and comparison of fragments longer than 300 bp [108].

### Homologous fragment analysis

We downloaded the *C. ensifolium* cp genome (MK841484.1) from the NCBI Organelle Genome Resources Database. Using BLAST (v2.2.25, https://blast.ncbi.nlm.nih.gov/Blast.cgi) software, we identified homologous genes and tRNA genes that were transferred from chloroplasts to mitochondria, with screening criteria of a matching rate of 70%, an E-value of 1e -5, and a length of 30 bp [108]. The results were visualized using Circos software (v0.69–5, http://circos.ca/software/download/) [109]. We also downloaded the assembled and annotated whole-genome data of *C. ensifolium* from the National Genomics Data Center under accession codes PRJCA005355/GWHBCII00000000. Using BLASTN (v2.10.1 +) software, we identified homologous genes that were transferred from mitochondria to the nucleus, with an E-value of 1e -5.

## Conclusions

In this study, we presented the first detailed characterization of a complete mitogenome in *C. ensifolium*. We assembled and annotated the mitogenome and thoroughly analyzed the DNA and amino acid sequences of annotated genes. The *C. ensifolium* mitogenome consisted of 19 circular chromosomes with a total length of 560,647 bp. We annotated 74 genes, including 35 PCGs, 36 tRNA genes, and 3 rRNA genes in the mitogenome. We also analyzed RNA editing sites, biased codon usage patterns, and repeat sequences. Furthermore, we analysed homologous fragments between mitochondria and chloroplasts, homologous fragments between mitochondria and the nucleus, Ka/Ks ratios, nucleotide polymorphism, synteny, PCG conservation in Orchidaceae and genomic features to gain a more comprehensive understanding of mitogenome evolution in Liliidae. Additionally, we verified the evolutionary status of *C. ensifolium* through phylogenetic analysis based on its mitogenomes and those of 29 other Liliidae plants. This comprehensive study provides valuable information on the *C. ensifolium* mitogenome, which will facilitate future research on species identification, genetic variation, and systematic evolution. In conclusion, these results contribute to the mitochondrial database of orchid plants and establish a detailed foundation for further research on this highly ornamental orchid.

### Supplementary Information

**Supplementary Material 1.**

## Data Availability

The raw sequencing data for the Illumina and Nanopore platforms and the mitogenome sequences have been deposited in NCBI (https://www.ncbi.nlm.nih.gov/) with accession numbers Nos. OR754263-OR754281.

## Abbreviations

ORFs: Open reading frames
PCGs: Protein-coding genes
rRNA: Ribosomal RNA
tRNA: Transfer RNA
CDS: Coding sequence
CMS: Cytoplasmic male sterility
HGT: Horizontal gene transfer
SSRs: Simple sequence repeats
RSCU: Relative synonymous codon usage
Ks: Synonymous mutation frequency
Ka: Nonsynonymous mutation frequency
Ka/Ks: Nonsynonymous-to-synonymous substitution ratio
